# Efficacy of 0.01% low dose atropine and its correlation with various factors in myopia control in the Indian population

**DOI:** 10.1038/s41598-022-10079-1

**Published:** 2022-05-02

**Authors:** Shweta Chaurasia, Seema Negi, Ashok Kumar, Srishti Raj, Sushmita Kaushik, Rahul Khanna M. Optom, Pranav Kishore, Mangat Ram Dogra

**Affiliations:** 1 Department of Ophthalmology, Advanced Eye Centre, Post Graduate Institute of Medical Education and Research, Sector 12, Chandigarh, India; 2 National Institute of Nursing Education, PGIMER, Chandigarh, India; 3 Department of Optometry, Advanced Eye Centre, Post Graduate Institute of Medical Education and Research, Sector 12, Chandigarh, India; 4 Centre of Sustainable Built Environment, MSAP, Manipal Academy of Higher Education, Manipal, India

**Keywords:** Drug screening, Phenotypic screening

## Abstract

We aimed to evaluate the efficacy and safety of low-dose atropine compared to placebo in the Indian population and also to study the impact of various modifiable and non-modifiable factors on myopia progression (MP) and drug efficacy (DE). It was a single-centre prospective placebo-controlled interventional study. 43 participants aged 6–16 years with progressive myopia received 0.01% atropine in the right eyes (treatment) and placebo in the left eyes (control) for 1-year. The main outcome measures were annual MP and axial length elongation (ALE) in treatment and control eyes and their percentage difference between two eyes (drug efficacy). Secondary outcome measures were the occurrence of any adverse events and the correlation of MP, ALE, and DE with various factors. 40 participants (80 eyes) completed the follow-up. After 1-year, MP was 0.25 D (IQR 0.13–0.44) and 0.69 D (IQR 0.50–1.0) (p < 0.001) in treatment and control respectively (63.89% reduction) with respective ALE of 0.14 mm (IQR 0.05–0.35) and 0.32 mm (IQR 0.19–0.46) (p < 0.001) (44.44% reduction). No adverse events were noted. Reduction in MP and ALE was statistically significant in all children irrespective of age-group, baseline MP, family history, screen-time, near and outdoor-time. The strongest determinants of annual MP were age (Treatment: r = − 0.418, p = 0.007; Control: r = − 0.452, p = 0.003) and baseline MP (Treatment: r = 0.64, p = 0.000; Control: r = 0.79, p = 0.000). Screen-time in control eyes was associated with greater ALE (r = 0.620, p = 0.042). DE was higher when outdoor time exceeded 2 h/day (p = 0.035) while the efficacy was lower with prolonged near activities (p = 0.03), baseline fast-progressors (p < 0.05) and history of parental myopia (p < 0.05). 0.01% atropine is effective and safe in retarding MP and ALE in Indian eyes.

## Introduction

Progressive myopia in growing children has become a major public health burden worldwide^1^ and its control remains the current priority and challenge. Topical atropine has emerged as the most effective and promising treatment modality in myopia control for over several decades^2–8^. Previous reviews and meta-analyses reported that among various treatment options available, topical atropine shows a maximum reduction in myopia progression (MP)^9,10^. Recently, different studies have shown that low-dose (0.01%) atropine has a better treatment to side-effect ratio and is an effective and safe treatment modality in myopia control when compared to its higher doses^7,11^. But to date, there is paucity in the literature concerning studies that have directly evaluated the lower concentration of atropine (0.01%) against a placebo^11^. Also, different studies on myopia control have subjected both eyes of individuals to low-dose atropine, and various confounding factors that vary from one individual to another and are known to cause an impact on MP were never considered^7,11^. Only one study has documented the results after matching the confounding factors (baseline near work, outdoor time, and accommodation)^11^. The effect of various confounding factors can be diminished if one eye of an individual receives the treatment and another eye of the same individual can act as the control and the difference in mean spherical equivalent (SE) between the two eyes can be used to calculate the true efficacy of the lowest dose of atropine in an individual. While an increase in urbanization, a decrease in outdoor activity^12–14^, and an increase in time spent on near work^15–18^ have been proposed to explain the recent increasing trends of myopia in children, it would be interesting to know with a given set of confounding factors in an individual, how this drug behaves compared to placebo. There is a published literature^5^ using atropine drops in one eye and the fellow eye as control, but the concentrations used were high and there is no study using 0.01% strength. Considering the existing paucity in the literature and non-availability of data from the Indian-sub-continent so far, our study aimed to study MP and axial length elongation (ALE) in the Indian population with one eye as treatment (0.01% atropine) and fellow as a control to evaluate the true efficacy of the drug in an individual. We also aimed the how various modifiable and non-modifiable factors impact MP, and how the efficacy of the drug differs in the setting of different factors.

## Methods

It was a single-center, prospective, interventional study conducted over one year in a tertiary-eye-care of north India. This trial was registered in the Chinese Clinical Trial Registry (ChiCTR2100051781) dated 04/10/2021 and followed the tenets of the Declaration of Helsinki. The study protocol received institutional (Post Graduate Institute of Medical Education and Research) review board approval. Clinical records of myopic children who underwent cycloplegic refraction at the institution in the past one year were screened and children aged 6–16 years with myopia ranging from − 1 D to − 7 D (SE) in both eyes, progression equal or greater than − 0.5 D in the preceding year, stable astigmatism of 1.5 D or less, anisometropia of 2 D or less, and best-corrected visual acuity at least 6/9 were enrolled in the study (selection and enrolment of the children being done until 31st October 2018). Patients with ocular pathology like spherophakia, retinal dystrophies, corneal dystrophy or other diseases, manifest strabismus, allergy to atropine eye drops, or children who were already under treatment for myopia control were excluded. Written informed consent was obtained from parents or guardians, and verbal consent was obtained from the participants. All participants received treatment with 0.01% atropine eye drops every night in the right eyes as the treatment group and 0.5% carboxymethyl cellulose drops in the left eyes as a control group for 1-year.

All participants underwent a detailed ophthalmological examination at the time of recruitment. Best-corrected visual acuity was measured with Snellen distance chart. Cycloplegic autorefraction was performed using an autorefractor (Huvitz, HRK-8000A Autorefractor-Keratometer). Optical biometry was performed using partial coherence interferometry based Optical Biometer (IOL Master 700, Carl Zeiss Meditec, Jena, Germany), and a mean of 3 readings was taken for axial length, lens thickness, anterior chamber depth. Pupil size was measured in both eyes using a scale in photopic conditions. Intraocular pressure was measured in both eyes using a non-contact tonometer. Hirschberg test and cover-uncover test were done to look for any manifest squint. The alternate cover test was performed to detect phoria for both distance and near and measured with a prism-bar cover test. The accommodation facility was checked using ± 2 Diopter flippers. Participants were allowed practice before the first test to ensure that they understood the test procedures Measurement of accommodation lag/lead was done by the monocular estimated method retinoscopy. Negative and positive relative accommodation and near the point of accommodation and convergence were also measured. The annual baseline rate of MP (BMP) in the child was calculated based on the cycloplegic refraction available in the documented previous-year data of the patient. A full refractive correction was prescribed to each participant during enrollment. Participants were followed up every 4-monthly up to one year (4th, 8th, and 12th month from recruitment). Cycloplegic refraction in terms of SE, photopic pupil size, and axial length were measured at each follow-up. Any change in the SE of ≥ 0.5 D on follow-ups was prescribed.

During enrollment, an assessment was done via a one-on-one interview conducted in the clinic via a structured validated questionnaire to obtain basic information regarding demography, parental history of myopia, and behavioral daily activities. Parental myopia was assessed by documenting the history of spectacles for distance in one or two parents. A parent was considered myopic if he or she had been using glasses for distance vision (minus lenses) before 18 years of age. Participants underwent assessment of their baseline day-to-day behavioral pattern in the most recent year such as the amount of time spent doing activities done at a short distance (near-work) apart from school hours (such as reading, writing, school assignments, drawing, craft-work, etc.), time spent on near gadgets (like smart phones, tablets, i-pads, laptop, video-games, etc.) and outdoor activities in daylight (outdoor sport, time spent in own backyard, going for walks, etc.).

At the time of enrollment, all participants were encouraged to refrain from smartphones and near gadget use (for gaming, movies, operation of social media) as part of lifestyle modification. They were counseled regarding the importance of sunlight exposure and were advised to indulge in outdoor activities for ≥ 2 h/day^19^ (preferably under diffuse day-light) and take a break of 1–2 min after every 20 min of near activity. The same questionnaire was filled up by the participants and their parents at all follow-ups for and the mean time spent near work, near gadgets, and outdoor activities were documented as hours per day (h/day). Any side effects and changes in pupillary diameter during the treatment were also noted.

The measure of change in SE refraction and axial length per year was the clinical relevant marker for the progression of SE (MP) and axial growth (ALE). True Efficacy of drug in an individual was described as a numerical annual reduction of MP and/or ALE in treatment eyes from control eyes and/or their percentage reduction calculated as below:

1. True reduction in MP (TRMP = MP _control eyes_ − MP _treatment eyes_) and
2. True reduction in ALE (TRALE = ALE _control eyes_ − ALE _treatment eyes_).
3. %TRMPD (percentage reduction in MP in treatment eyes compared to control) = TRMP × 100/MP _control eyes_.
4. %TRALE or percentage reduction in ALE in treatment eyes compared to control) = TRALE × 100/ALE _control eyes_.

### Statistical analysis

Statistical analysis was done by IBM SPSS software V 22.0. Data were tabulated and compared between the two eyes of each patient. Categorical variables were described as proportions. Shapiro Wilk Test, Histogram, and Q–Q plot were applied to check the normal distribution of continuous data. Normally distributed continuous variables were described as mean and standard deviation whereas skewed data was described as median and IQR. Wilcoxon signed-rank test was applied to compare the median difference of MP between treatment and control eyes in factors (with quantitative data) whereas depending on different factors like age, near work, digital time, outdoor time, BMP, and family history, children were categorized in two sub-groups. Mann–Whitney *U*-test was employed to compare the median difference of MP and ALE between the subgroups. Spearman’s rank correlation coefficient to find out the significant correlation between MP and ALE and various factors.

### Conference presentation

WOC 2020, WSPOS 2020.

## Results

A total of 104 subjects were assessed for eligibility, and 43 subjects were recruited into the study but 80 eyes of 40 children (M/F:20/20) with progressive myopia were analyzed (3 children lost follow-up). The mean age was 11.83 ± 2.35 years (6-16 years). The distributions of children aged between 6–11 years and 12–16 years were 14(35%) and 26(65%) respectively. Out of a total of 40 children, 13 children (32.5%) in our study had a family history of myopia (either of their parents).

### Baseline parameters (Table 1)

**Table 1:** Average of baseline parameters in treatment and control eyes (before the start of study).

Baseline parameter	Treatment group i.e. right eyes	Control group i.e. left eyes	p value
	Mean ± SD	Mean ± SD	
Baseline cycloplegic refraction in terms of Spherical equivalent (D)	− 3.04 ± 1.36	− 3.07 ± 1.32	0.733
K1 (D)	43.78 ± 1.46	43.83 ± 1.39	0.301
K2 (D)	44.66 ± 1.47	44.76 ± 1.44	0.072
Axial length (mm)	24.52 (23.91–25.07)	24.56 (23.92–25.02)	0.416
Lens thickness (mm)	3.38 (3.19–3.49)	3.37 (3.21–3.53)	0.765
Anterior chamber depth (mm)	3.82 (3.50–4.03)	3.81(3.49–4.03)	0.898
Near Phoria (pd BI) (n = 21/40)	2.40 ± 2.83		
Distance phoria (pd BI) (n = 29/40)	1.45 ± 2.63		
NPC/NPA	7.58 ± 2.01/ 9.40 ± 1.71		
PRA	− 3.20 ± 1.05		
NRA	2.45 ± 0.37		
Accommodation lag	0.56 ± 0.32	0.58 ± 0.31	0.589
Pupil size (mm)	3.18 ± 0.37	3.18 ± 0.38	1.000
Accommodation facility (cpm)	10.65 ± 1.51		
IOP (mmHg)	14.15 ± 1.72	14.65 ± 1.78	0.068
Screen time (h/day)	1.11 ± 0.72		
Near work (h/day)	3.11 ± 0.96		
Outdoor (h/day)	1.23 ± 0.66		
Baseline Myopia Progression (D)	0.89 ± 0.26		

*D* dioptre, *K* keratometric reading, *PBCT* prism bar cover test, *pd*prism dioptre, *BI* base in, *NPC* near point of convergence, *NPA* near point of Accomodation, *NRA* Negative relative accommodation, *PRA* positive relative accommodation, *cpm* cycles per minute, *IOP* intra-ocular pressure.

The mean baseline SE, baseline axial length (AL), baseline MP, pupil size, and of other biometric parameters in right eyes (treatment group) and left eyes (control group) of 40 children have been shown in Table 1. Accommodation lag, NRA, and PRA of all patients were within normal limits (Table 1) but the accommodation facility was on the lower side (mean 10.65 ± 1.51cycles/min). After full correction of myopia, 21(52.5%) children had orthophoria, 19 (47.5%) had exophoria (mean 2.40 ± 2.83 prism diopter base in) and none had esophoria.

### Parameters studied

**Figure 1:**
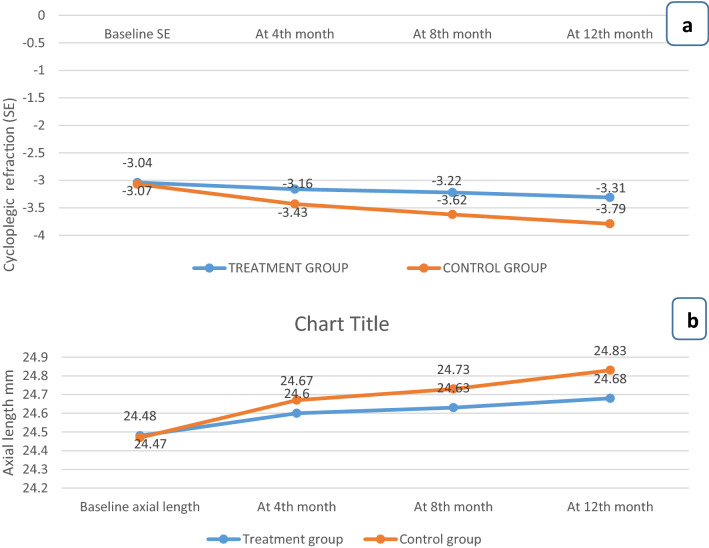
Time series plot depicting Spherical equivalent (SE) and Axial length over 1-year period: (**a**) Comparison of change in cycloplegic refraction (in terms of SE) in treatment group and control group during study period. (**b**) Comparison of axial length in treatment group and control group during study period.

At the end of the study, mean SE and mean AL in treatment (T) eyes have been shown in Table 2. Figure 1 shows mean SE and AL in treatment and control (C) eyes at different follow-ups.

**Table 2:** Average of studied parameters (after the completion of 1-year) in treatment and control eyes.

Parameter during study period	Treatment group i.e. right eyes	Control group i.e. left eyes	p value
	Mean (± SD)	Mean (± SD)	
Pupil size (mm)	3.86 ± 0.47	3.18 ± 0.37	0.0001
MP (D)	0.26 ± 0.23	0.72 ± 0.29	0.0001
ALE (mm)	0.20 ± 0.21	0.36 ± 0.24	0.0001
TRMP (D)	0.46 ± 0.21		
TRALE (mm)	0.16 ± 0.11		
Percentage TRMP between two eyes (%)	66.34 ± 21.21		
Percentage TRALE between two eyes (%)	54.01 ± 27.66		
Screen-time (h/day)	0.31 ± 0.58		
Near work duration (h/day)	3.28 ± 0.94		
Outdoor time (h/day)	2.39 ± 0.40		

*D* Diopter, *mm* millimeters, *MP* Myopia progression, *ALE* axial length elongation, *TRMP* True reduction in Myopia progression in treatment eyes compared to control, *TRALE* True reduction in ALE in treatment eyes compared to control, *%TRMP* percentage TRMP, *%TRALE* percentage TRALE.

#### MP (total change in SE from baseline to the last follow-up)

After 1-year mean MP in 40 treatment eyes was 0.26 ± 0.23 Diopter (D) and 40 control eyes was 0.72 ± 0.29 D and the difference between the two was statistically significant (p < 0.001) (Table 2).

#### ALE (total change in AL from baseline to the last follow-up)

ALE at the end of 1-year in treatment and control eyes was 0.20 ± 0.21 mm and 0.36 ± 0.24 mm respectively and their difference was statistically significant (p < 0.001) (Table 2).

#### Comparison of MP and ALE between treatment and control eyes among various sub-groups (e-Table 5)

Difference between average MP and ALE in treatment and control eyes in sub-groups of various factors (screen-time, near-work time, outdoor-time, age, BMP, family history was statistically significant (p < 0.001).

#### True efficacy and percentage true efficacy of the drug (TRMP, TRALE, %TRMP, %TRALE)

Compared to placebo eyes, there was a reduction of 0.46 ± 0.21 D or 66.34 ± 21.21% of MP and 0.16 ± 0.11 mm or 54.01 ± 27.66% of ALE in treatment eyes (Table 2).

#### Safety profile of low-dose atropine

Photopic pupil size in treatment eyes and control eyes was 3.86 ± 0.47 mm and 3.18 ± 0.37 mm respectively (p = 0.0001) (Table 2). But no patient complained of allergy to atropine drops, blurring of vision, photophobia, or glare.

### Effect of various modifiable and non modifiable factors on mp and ALE

#### Digital screen-time

**Figure 2:**
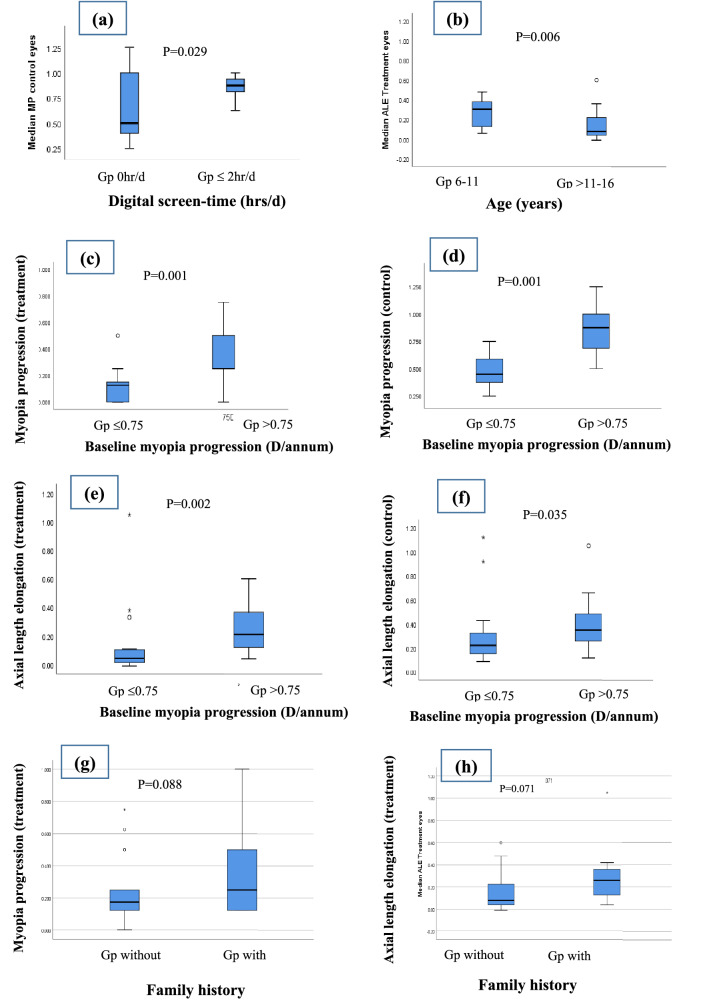
Box-whisker-plots showing the difference of MP or ALE between sub-groups: (**a**) MP (control eyes) in children not using digital device vs children with screen time upto 2 h/day (**b**) ALE (treatment eyes) in children 6–11 years vs > 11–16 years; (**c**) MP (treatment eyes) in children with BMP ≤ 0.75 D/annum versus BMP > 0.75 D/annum; (**d**) MP (control eyes) in children with BMP ≤ 0.75 D/annum versus BMP > 0.75D/annum; (**e**) ALE (treatment eyes) in children with baseline myopia progression ≤ 0.75 D/annum vs > 0.75 D/annum; (**f**) ALE (control eyes) in children with baseline myopia progression ≤ 0.75 D/annum vs > 0.75 D/annum; (**g**) MP (treatment eyes) in children without and with family history; (**h**) ALE (treatment eyes) in children without and with family history. (*H/d* hours/day, *MP* myopia progression, *ALE* axial length elongation, *D/annum* Dioptre per annum, *BMP* baseline myopia progression).

**Figure 3:**
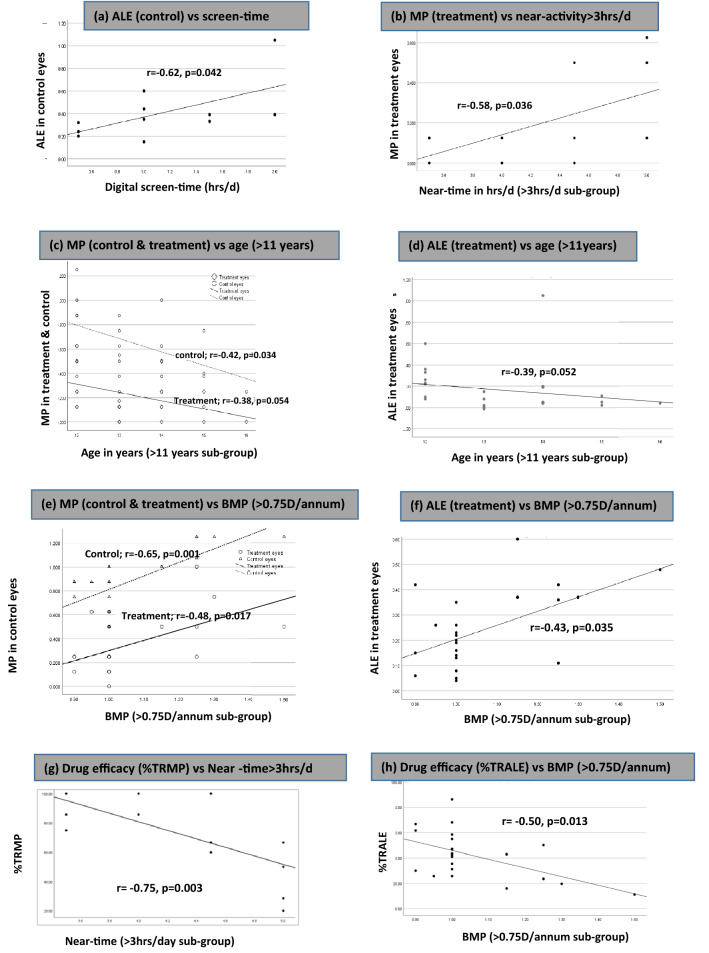
Scatter plot showing correlation of MP, ALE, %TRMP and % TRALE with various sub-groups of modifiable and non-modifiable factors: (**a**) correlation of ALE (control eyes) vs screen time in children using digital devices (upto 2 h/d); (**b**) correlation of MP (treatment eyes) vs near-time in children with near activities > 3 h/d; (**c**) correlation of MP (control and treatment eyes) with age in children > 11 years; (**d**) correlation of ALE (treatment eyes) vs age in children > 11 years; (**e**) MP (treatment and control eyes) versus BMP in children with baseline fast progression (> 0.75 D/annum); (**f**) ALE (treatment eyes) versus BMP in children with fast progression (> 0.75 D/annum); (**g**) %TRMP versus near-time in children spending > 3 h/d; (**h**) %TRALE versus BMP in children with fast progression (> 0.75 D/annum). *MP* myopia progression, *ALE* axial length elongation, *h/d* hours per day, *%TRMP* percentage total reduction in myopia progression, *%TRALE* percentage total reduction in axial length elongation, *D* dioptre, *BMP* baseline myopia progression).

29/40 children did not use near gadgets while rest used them for variable time up to 2-h daily. Children who didn’t use digital devices had annual MP of 0.18D in treatment eyes and 0.50 D in control eyes whereas children who used digital devices upto 2 h/day had MP of 0.25D in treatment eyes and 0.88D in control eyes (e-Table 5). Statistically, average MP was significantly higher in control eyes of children using digital devices compared to the ones not using at all (p = 0.029; Fig. 2a). There was statistically significant weak positive correlation between screen-time and MP in control eyes of all 40 children (r = 0.37, p = 0.02; Table 3) but strong positive correlation between screen-time and ALE in control eyes of children using digital devices (r = 0.62, p = 0.042; Fig. 3a).

**Table 3:** Correlation of MP and ALE of treatment and control eyes in all 40 children.

Various factors n = 40	MP (Myopia progression)		ALE (Axial length elongation)	
	Treatment eyes r value (p value)	Control eyes r value (p value)	Treatment eyes r value (p value)	Control eyes r value (p value)
Digital screen-time (modifiable)	0.24 (0.129)	0.37 (0.021)*	0.18 (0.272)	0.23 (0.150)
Near-time (modifiable)	− 0.10 (0.524)	− 0.17 (0.308)	− 0.13 (0.433)	− 0.17 (0.284)
Outdoor-time (modifiable)	− 0.21 (0.201)	− 0.09 (0.578)	− 0.23 (0.164)	− 0.16 (0.319)
Age (non-modifiable)	− 0.42 (0.007)*	− 0.45 (0.003)*	− 0.53 (0.000)*	− 0.42 (0.007)*
Baseline myopia progression (non-modifiable)	0.64 (0.000)*	0.79 (0.000)*	0.56 (0.000)*	0.43 (0.006)*

*p value of statistical significance < 0.05.

#### Time spent on near work and outdoor activity

There was no correlation between MP and near-work time in control eyes of 40 children (r = − 0.17, p = 0.308; Table 3). But there was a moderate positive correlation between MP and near-work time in treatment eyes of children spending > 3 h/day (r = 0.58, p = 0.036; Fig. 3b). There was no overall significant correlation of MP and ALE with outdoor time in our study (Table 3).

#### Age

Overall results of our study showed a moderate negative correlation of age with MP and ALE in treatment and control eyes of 40 children (MP: r = − 0.42, p = 0.007; r = − 0.45, p = 0.003; ALE: r = − 0.53, p = 0.000; r = − 0.42, p = 0.007 respectively) (Table 3). Among the two age groups (6–11 vs > 11 years), negative correlation of age with MP was greatest in control eyes (C) of older children (r = − 0.42, p = 0.034; Fig. 3c). Treatment eyes (T) in older children also showed a trend towards decline in MP and ALE with progressive age (r = − 0.38, p = 0.054; r = 0.39, p = 0.052 respectively; Fig. 3c,d). Also, annual axial growth of treatment eyes was faster in younger children compared to older ones (0.31 mm vs 0.08 mm p = 0.006; Fig. 2b).

#### BMP

There was overall significant moderate-strong positive correlation of MP and ALE with BMP in both treatment (T) and control (C) eyes [MP(T): r = 0.64 (p = 0.000), MP (C): r = 0.79 (p = 0.000); ALE(T): r = 0.56 (p = 0.000), ALE(C): r = 0.42 (p = 0.000); Table 3]. This positive correlation was clinically significant in fast progressors (BMP > 0.75 D/annum) both in the treatment and control eyes [MP(T): r = 0.48 (p = 0.017), MP(C): r = 0.65 (p = 0.001); ALE (T): r = 0.43 (p = 0.035) Fig. 3f–h]. The difference of MP and ALE between fast and slow progressors was clinically significant both in the treatment and control eyes [MP(T): p = 0.001; MP(C): p = 0.001; ALE(T): p = 0.002; ALE(C): p = 0.035; Fig. 2c–f].

#### Family history

There was no statistical difference in MP or ALE in control eyes between children with family history and without a family history (etable). There was a trend towards lesser MP and ALE in treatment eyes of children without family history compared to children with family history (p = 0.088 and 0.071 respectively; Fig. 2g,h).

### Effect of various modifiable and non modifiable factors on drug efficacy

#### Digital screen time

No correlation was seen between %TRMP and %TRALE and screen-time up to 2-h/day in all 80 eyes (Table 4).

**Table 4:** Correlation of drug efficacy (TRMP, TRALE, %TRMP, %TRALE) with various modifiable and non-modifiable factors.

Various factors	Drug efficacy			
n = 40	TRMP r value (p value)	TRALE r value (p value)	%TRMP r value (p value)	% TRALE r value (p value)
Digital screen-time (modifiable)				
Up-to 2 h/day	0.25 (0.115)	0.15 (0.372)	− 0.11 (0.499)	− 0.15 (0.360)
Near-time (modifiable)				
	− 0.23 (0.147)	− 0.17 (0.301)	− 0.02 (0.924)	0.05 (0.771)
Outdoor-time (modifiable)				
	0.19 (0.240)	0.16 (0.320)	0.26 (0.099)**	0.28 (0.083)**
Age (non-modifiable)				
6-16 years	− 0.25 (0.118)	0.02 (0.925)	0.22 (0.164)	0.42 (0.007)*
BMP (non-modifiable)				
	0.48 (0.002)*	− 0.14 (0.390)	− 0.37 (0.018)*	− 0.59 (0.000)*

*p value of statistical significance < 0.05; **p value showing trend for significance though not statistically significant. *TRMP* True reduction in Myopia progression in treatment eyes compared to control; *TRALE* True reduction in Axial length elongation treatment eyes compared to control, *%TRMP* percentage TRMP, *%TRALE* percentage TRALE.

#### Near work, outdoor activity

There was no correlation of %TRMP and %TRALE with near work in all 40 children (Table 4) but a strong negative correlation of near-time with %TRMP was seen in group of children (n = 13/40) spending > 3 h daily in near activities (r = − 0.75, p = 0.003; Fig. 3g).

**Figure 4:**
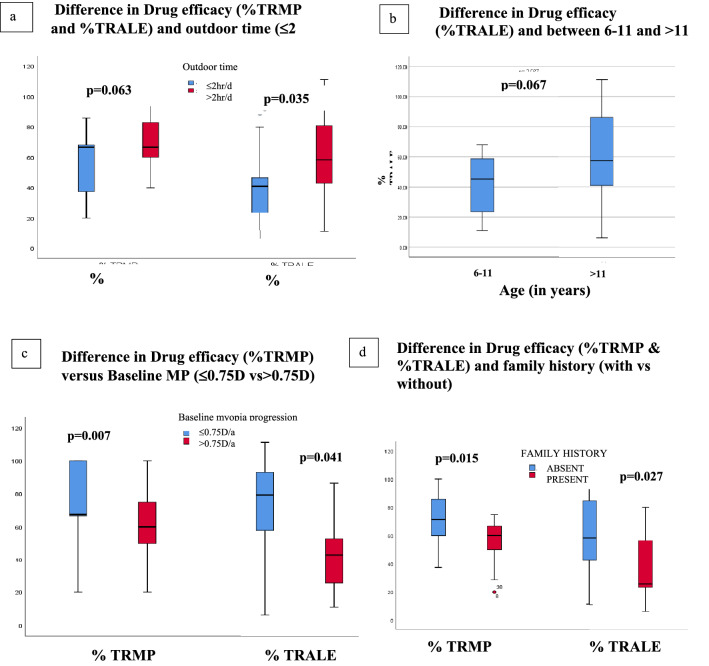
Box-whisker-plots showing the difference of %TRMP and %TRALE between groups: (**a**) with outdoor ≤ 2 h/day vs > 2 h/day; (**b**) age 6–11 years vs > 11 years (**c**) BMP) ≤ 0.75 D/annum vs > 0.75 D/annum (**d**) with family history vs without family history. (*D* Dioptres, *BMP* baseline myopia progression, *%TRMP* Percentage reduction in Myopia progression in treatment eyes compared to control eyes, *%TRALE* percentage reduction in axial length elongation in treatment eyes compared to control eyes).

Drug efficacy (%TRMP and %TRALE) showed a borderline weak correlation with outdoor-time in 40 children (Table 4). But the difference in %TRALE between children with outdoor time ≤ 2 h/day and > 2 h/day was statistically significant (p = 0.035; Fig. 4a).

#### Age

%TRALE in treatment eyes showed moderate positive correlation with age (r = 0.42; p = 0.007; Table 4). Compared to placebo the drug was equally effective (in terms of %TRMP) in younger as well as older children as there was no significant difference in %TRMP between the two subgroups of age (%TRMP 6–11 vs > 11 years: 66.63% vs 66.67%, p = 0.511). But difference of % TRALE between older and younger subgroup of children was borderline clinically significant 57.43% vs 45.22% p = 0.067; Fig. 4b).

#### BMP

There was a weak to moderate negative significant correlation between drug efficacy and BMP in 40 children (BMP vs %TRMP: r = − 0.37, p = 0.018; BMP vs %TRALE: r = − 0.59, p = 0.000; Table 4). Among the two sub-groups, fast myopic progressors (> 0.75 DS/annum) showed significant moderate negative correlation of BMP with %TRALE (r = − 0.50, p = 0.013; Fig. 3h). Also, there was significant difference of TRMP, %TRMP and %TRALE between children with BMP ≤ 0.75 D/annum and BMP > 0.75 D/annum (p-value: 0.007, 0.041, 0.01 respectively; Fig. 4c).

#### Family history

There was a significant difference in TRMP, %TRMP and %TRALE between children with a family history or without a family history (family history present vs absent: MP, p = 0.039; %TRMP, p = 0.015; %TRALE, p = 0.027; Fig. 4d).

## Discussion

The results of our study showed that a once-nightly dose of 0.01% atropine eye drops achieved a statistically and clinically significant reduction in MP and ALE in low and moderate childhood myopia compared with placebo treatment in Indian eyes. Compared to the results of meta-analysis^20^ of various randomized clinical trials and cohort studies using varying atropine concentrations from 1 to 0.05%, the average difference in SE between treated and control eyes in our Indian children (0.46 D/year) using a much lower concentration of atropine was comparable to the published results of mixed concentrations in Asian children and greater than Caucasian children^20^. Compared to placebo, 0.05% atropine group of LAMP study^11^ showed a reduction of 67% of MP and 51% of AL elongation which was comparable to the results of our study using 0.01% atropine in 6–12 years age-group population. While comparing the efficacy, it is important to note that the mean MP of placebo eyes of LAMP) study (0.81 ± 0.53 D) was similar to mean MP of placebo eyes of our study in 6–12 years age group (0.81D). Though in the second year, of LAMP study^21^, 0.01% atropine was mildly more effective than its first year (0.48 ± 0.44 D, 0.25 ± 0.18 mm) but its efficacy was still lesser than our study. The probable reason could be younger age (4–12 years), ethnicity and competitive educational environment and urbanized lifestyle of East Asians compared to our Indian population.

## Relationship between myopic progression and various factors

### Modifiable

Several studies^16,18,19,22–24^ have examined the individual relationship of digital screen-time as a risk factor for myopic prevalence. But physiological aspects of screen exposure translating into myopia-related outcomes are not clear and consistent evidence of an association between screen-time and myopia prevalence and progression is lacking^19,22–24^. Recent studies have agreed on a trend of increasing myopia with increased screen-time^18,25–27^. In our study, all children who were enrolled in our study were counseled not to use near gadgets. 72% of children were compliant while others only reduced screen-time but no child used it beyond 2 h/day during the study period. In the absence of treatment, MP and axial growth were greater in children who spent screen-time with near-gadgets. Compared to children who were compliant enough to refrain from using digital devices, children who used digital devices had an additional annual MP of 0.38D in control eyes but only 0.08D in treatment eyes. Though the values do not seem clinically significant on the annual follow-up, but additional increments in MP with increased screen-time in absence of treatment may cumulate to a significant value over long period of time and future studies with long follow-ups can substantiate the same. Hence, the use of near smart-gadgets must be discouraged where ever possible.

Many authors have investigated near-work activity as an independent risk factor for myopia, however, studies of the associations between myopia and near-work activities have produced less consistent results^22,28–30^. Increase of near-work activity is interlinked with the concomitant reduction of outdoor activities^31,32^ and the direct and independent implication of near-work time with myopia can be difficult. Many authors reported high near-work activities to be related to higher odds of myopia development and progression^18,32,33^. By contrast, other studies reported no significant correlation between near-work activities (even > 3 h/day) and myopia^13,34^. In our study, the median near work time was 3 h/day (range 1.5–5 h/day) apart from school. We found no association of MP with near work in control eyes (including children doing prolonged near activities > 3 h/day. This could be because all children were counseled/encouraged to read at > 30 cm and take brief breaks while doing any near-work as a protective measure against extra-hour of near-work and all children fairly followed the advice in a year follow-up. Another reason could be that small number of children (5 children) indulged in extended duration of near activities (5-h/day).

In our study, we did not find any association of MP and ALE with time spent outdoors in control eyes. Read et al.^35^ concluded that associations between myopia and outdoor activity are likely due to exposure to bright outdoor light rather than greater physical activity. In our study, children were counseled to indulge in outdoor activities for > 2 h per day^18^ and 68% were fully compliant while the rest also engaged in some amount of outdoor activity with day-light exposure. Various studies have demonstrated that outdoor time bears a protective role in reducing the onset of myopia, rather than slowing down the progression of the disease^14,34,36–38^. The Anyang Childhood Eye Study also revealed that children who were already myopic at baseline did not show any association between outdoor-time and reduction of axial elongation^38^. These differences indicate that factors that affect the risk of myopia onset may differ from those that affect the rate of MP.

### Non-modifiable

MP in the placebo eyes among the youngest population (6–9 years) in our study (0.92 ± 0.33 D) was overall similar to 6–7 years of children of COMET during 1st year of study (0.87 D)^39^ but higher than their 8- and 9-years age-group (0.65 DS and 0.54 DS respectively). Baseline age was the strongest independent factor for myopia progression and axial length growth both in presence or absence of treatment. Like other studies^39,40^ results of our study also showed that MP was faster in younger children than older ones. This protective impact of increasing age on slowing down MP was maximal in older children. Also, in presence of drug, annual axial growth of olden children remained substantially arrested. Similarly with progressing age, the rate of axial-growth declined among both atropine-treated and control subjects. Hence, lowest strength atropine may work effectively in older children but children with myopia at a younger age need to be monitored closely as efficacy of low dose atropine might not match their faster rate of progression.

Annual BMP (before the start of the study) was another important non-modifiable predictor of myopia in our study. 50% of our study population showed BMP < 1 D. Rapid baseline myopic shift (> 1 DS) was seen in 17.5% of children in our study as against 7.4% in the NIM study^41^. Like other studies, younger age, and children with greater severity of initial myopic refraction were found to have faster myopia^42^. In our study, the average age, and SE of children with BMP > 1 D were 11.50 years and − 3.50 DS respectively. Annual rate of MP calculated in our study correlated well with its BMP (before the start of the study) in control eyes and decreased substantially compared to its baseline value in treatment eyes (e-Table 5). So, knowing the baseline value of progression in a child before the start of treatment may help in the prognostication of real-time future outcomes.

Placebo eyes in our study had MP of 0.69 DS which was comparable to Asian children in COMET^39^. Compared to the BMP (0.98 D), there was a drop-in rate of MP in placebo eyes after 1-year and the reason could be either dampening effect of age progression on MP or protective effect of lifestyle modifications re-enforced via counseling throughout the study including breaks in near activities, decrease in screen time and increase in outdoor time in sunlight (Tables 1 and 2) or their combined effect.

The link of heritability to myopia has been substantiated by many epidemiological and genetic studies^43–45^. Many studies have found a higher risk of developing myopia in children with two myopic parents compared to children with no myopic parents^12,19,43,44^. However, heritability studies can project overestimations and it does not establish a compulsory relationship as members of the same family often share similar environmental and lifestyle factors^45^. Our study did not find any association of family history with MP. Non-association of parental history with MP found in our study could be because all children in our study had myopia in one of the parents who were found to be low myopic with onset in late teens. Also, it could be because the discovery of several genetic loci has related the parental (family) history of myopia with the onset of myopia, rather than the progression of myopia^43,44,46–48^.

In our patients, baseline accommodative lags of all children fell within the normal range and none of the patients had esophoria as found in other studies^49^. So, our study can’t reliably support the accommodation lag and esophoria as causative proponents of progressive myopes^19,43–45,50,51^. But transient accommodation errors or abnormal responses to blur might be suggested as one of the causative factors or associated findings in these progressive myopes as lower levels of accommodation facility^52^ were found in our study.

## Relationship between drug efficacy and various factors

The impact of environmental modifiable and non-modifiable factors on drug efficacy has never been investigated. In our study**,** no impact of screen-time was seen on drug efficacy till the screen-time was limited to 2 h daily. Results of our study also showed that compared to placebo, low-dose atropine was equally effective in halting MP and axial-growth in all eyes receiving the drug irrespective of screen-time and duration of near-work but its efficacy proportionally decreased in children with high near demand (MP vs > 3 h/day: r = 0.58, p = 0.036) and hence, such children should be monitored closely. Also, future studies on the relationship of drug efficacy with screen-time and near-work can give more insight. The drug was also equally effective in halting myopia progression in all children indulging in outdoor activity (≤ 2 h/day vs > 2 h/day) but the drug had a more protective role in preventing axial growth of eyes in children with greater daily outdoor light exposure > 2 h/day compared to ≤ 2 h/day (%TRALE p = 0.035).

Compared to placebo the drug was equally effective in younger as well as older children in halting myopia progression though maximal effectivity of drug was seen in arresting axial growth of older children. Hence, it is prudent to believe that low-dose atropine can substantially decrease myopic burden in progressive myopes if started early and can provide a substantial additive protective role in halting axial growth of the eye in older children. Compared to placebo the drug was effective in all children with mean baseline annual MP ranging from 0.5 D to 1.5 D but the efficacy of low dose atropine in control of myopia particularly axial growth was significantly greater in slow progressors compared to fast progressors (≤ 0.75 D vs > 0.75 D: %TRMP-67.43% vs 60%, p = 0.041; and %TRALE-79.13% vs 42.66%, p = 0.010; Fig. 4c). So, it’s important to estimate baseline MP in a child before starting treatment in order to have real-time expectation from the treatment regime. Also, one should be careful using lowest strength atropine in fast progressors as the limited efficacy of this low-strength drug may add ocular morbidity over years. All children (both with and without a family history) responded well to treatment with 0.01% atropine drops but the efficacy of the drug was higher in children without a family history of myopia.

In a meta-analysis, Gong et al.^53^ found that the side effects of atropine drops are dose-related and seen very less often with low dose atropine (0.01%). In our study, eyes receiving 0.01% atropine had a significant change (0.50 mm) in photopic pupil size, compared to eyes receiving placebo (p = 0.0001). Change in pupil size in Indian eyes was comparable to pupil size in LAMP study^11^ (0.50 mm) and lesser than ATOM2 study^7^ (1.15 mm). But in our study, 0.01% atropine was well tolerated and no children complained of blurring of vision, photophobia, or any other side effect during follow-up visits.

The strength of the study was analyzing the true efficacy of the lowest strength of the drug in Indian eyes compared with placebo in the same individual, thereby, reducing confounding factors (genetic, environmental, and lifestyle-related). Also, the role of the drug in different individuals with varying confounding factors known to impact MP was considered and studied. Limitations of the study were small sample size and short follow-up. Also, a questionnaire-based system of data collection regarding risk factors has inherent limitations and may underestimate actual near, outdoor and screen-time. The relationship of near-work and outdoor time with MP and drug efficacy found in our study might not be real-time data as temporal factors considered in our study (e.g., break-time between texts which is an ideal situation) and prior counseling regarding lifestyle modification might have influenced results.

As age and MP were negatively correlated. Therefore, we can’t rule out the confounding effect on each other.

So, to conclude low dose atropine (0.01%) is effective and safe in Indian eyes for myopia control without any visual side-effects. Compared to placebo, low-strength of the drug showed good efficacy irrespective of age, screen-time, time spent indoor and outdoor, and family history but young age and fast progressors may still need close follow-up. These various modifiable and non-modifiable factors discussed in this manuscript gave an insight into their role in affecting the efficacy of the drug and future studies are warranted for understanding the same. Recommendations should be made to start the drug earliest in children with progressive myopia along with lifestyle modification of increasing outdoor-time, limiting indoor and screen-time, and encouraging regular breaks with near activities.

## Supplementary Information

Supplementary Tables.
