# Supplementary material for: Efficacy of 0.01% low dose atropine and its correlation with various factors in myopia control in the Indian population
Source: Sci Rep. 2022 May 2;12:7113. doi: 10.1038/s41598-022-10079-1 (PMC9061826; doi:10.1038/s41598-022-10079-1)
Supplement: Supplementary file 1 — Supplementary Tables. [file 41598_2022_10079_MOESM1_ESM.docx]

| **Sub-groups** | **MP in t/t**  **Eyes (a) Median**  **(IQR) in D** | **MP control eyes (b) Median**  **(IQR) in D** | **P value**  **a vs b** | **ALE Treatment eyes (x)**  **Median**  **(IQR) in mm** | **ALE control eyes (y) Median**  **(IQR) in mm** | **P**  **value**  **x vs y** |
| --- | --- | --- | --- | --- | --- | --- |
|  |  |  |  |  |  |  |
| **Digital screen-time** | | | | | | |
| Sub-Gp1(0 hr/d)  (29/40) | 0.175 (0.13-0.25) | 0.50 (0.388-1.0) | **0.000*** | 0.11 (0.05-0.34) | 0.29(0.18-0.47) | **0.000*** |
| Sub-Gp2 (≥2hr/d)  (11/40) | 0.25 (0.13-0.50) | 0.88 (0.75-1.0) | **0.003*** | 0.22 (0.08-0.37) | 0.35 (0.24-0.44) | **0.003*** |
| **Near-work time** | | | | | | |
| Sub-Gp 1 (3hrs/d)  (27/40) | 0.25 (0.13-0.25) | 0.748 (0.50-1.0) | **0.000*** | 0.16 (0.08-0.37) | 0.33 (0.22-0.50) | **0.000*** |
| Sub-Gp2 (≥3 hrs/d)  (13/40) | 0.13 (0.06-0.50) | 0.63 (0.38-0.94) | **0.001*** | 0.05 (0.04-0.23) | 0.29 (0.16-0.37) | **0.001*** |
| **Outdoor-time** | | | | | | |
| Sub-Gp1 (≤2hrs/d)  (13/40) | 0.25 (0.13-0.56) | 0.875 (0.46-1.0) | **0.001*** | 0.26 (0.07-0.42) | 0.39 (0.17-0.58) | **0.001*** |
| Sub-Gp2 (>2hrs/d)  (27/40) | 0.25 (0.13-0.25) | 0.63 (0.50-1.0) | **0.000*** | 0.11 (0.04-0.22) | 0.31 (0.22-0.39) | **0.000*** |
| **Age** | | | | | | |
| Sub-Gp1 (6-11yrs)  (14/40) | 0.25 (0.22-0.5) | 0.81(0.5-1.12) | **0.001*** | 0.31(0.13-0.39) | 0.45(0.23-0.56) | **0.001*** |
| Sub-Gp2 (>11-16yrs)  (26/40) | 0.13 (0.13-0.31) | 0.63 (0.38-0.88) | **0.000*** | 0.08 (0.04-0.22) | 0.27 (0.15-0.36) | **0.000*** |
| **Baseline Myopia Progression** | | | | | | |
| Sub-Gp1 (≤0.75D)  (16/40) | 0.13 (0.00-0.16) | 0.45 (0.38-0.61) | **0.000*** | 0.05 (0.01-0.11) | 0.23 (0.15-0.33) | **0.000*** |
| Sub-Gp2 (>0.75)  (24/40) | 0.25 (0.25-0.50) | 0.88 (0.66-1.0) | **0.000*** | 0.21 (0.12-0.37) | 0.35 (0.24-0.49) | **0.000*** |
| **Family history** | | | | | | |
| Sub-Gp1 Present (13/40) | 0.25 (0.13-0.50) | 0.625 (0.44-1.0) | **0.001*** | 0.26 (0.12-0.37) | 0.35 (0.23-0.49) | **0.001*** |
| Sub-Gp2 Absent (27/40) | 0.175 (0.13-0.25) | 0.75 (0.50-1.0) | **0.000*** | 0.08 (0.04-0.23) | 0.29 (0.17-0.39) | **0.000*** |

**e-Table 5**: Comparison of MP and ALE of treatment and control eyes within two subgroups of various modifiable and non-modifiable factors. * show p value of statistical significance <0.05; **p value showing trend for significance though not statistically significant. D-Diopter; Gp=group; hrs/d=hours/day; diff=difference; MP=myopia progression; ALE=axial length elongation.

**eTable 3**
